# Einsamkeit während der ersten Welle der SARS-CoV-2-Pandemie – Ergebnisse der NAKO-Gesundheitsstudie

**DOI:** 10.1007/s00103-021-03393-y

**Published:** 2021-07-29

**Authors:** Klaus Berger, Steffi Riedel-Heller, Alexander Pabst, Marcella Rietschel, Dirk Richter, NAKO-Konsortium, Wolfgang Lieb, Anne Hermes, Heiko Becher, Nadia Obi, Kathrin Günther, Wolfgang Ahrens, Stefanie Castell, Yvonne Kemmling, André Karch, Nicole Legath, Börge Schmidt, Carina Emmel, Oliver Kuß, Tamara Schikowski, Lena Koch-Gallenkamp, Bernd Holleczek, Antje Damms-Machado, Karin Halina Greiser, Karin B. Michels, Claus-Werner Franzke, Annette Peters, Sigrid Thierry, Beate Fischer, Michael Leitzmann, Markus Löffler, Kerstin Wirkner, Rafael Mikolajczyk, Dan Rujescu, Sylvia Gastell, Matthias B. Schulze, Lilian Krist, Julia Fricke, Lina Jaeschke, Tobias Pischon, Claudia Meinke-Franze, Henry Völzke

**Affiliations:** 1 Institut für Epidemiologie und Sozialmedizin, Universität Münster, Domagkstr. 3, 48149 Münster, Deutschland; 2 Institut für Sozialmedizin, Arbeitsmedizin und Public Health (ISAP), Medizinische Fakultät, Universität Leipzig, Leipzig, Deutschland; 3 Abteilung Genetische Epidemiologie in der Psychiatrie, Zentralinstitut für seelische Gesundheit, Mannheim, Deutschland; 4 Zentrum Psychiatrische Rehabilitation, Universitäre Psychiatrische Dienste Bern, Bern, Schweiz; 5 Universitätsklinik für Psychiatrie und Psychotherapie, Universität Bern, Bern, Schweiz; 6 Departement Gesundheit, Berner Fachhochschule, Bern, Schweiz

**Keywords:** Einsamkeit, Psychische Gesundheit, Depression, Angst, Kohortenstudie, Loneliness, Mental health, Depression, Anxiety, Cohort study

## Abstract

**Hintergrund:**

Mit Beginn der SARS-CoV-2-Pandemie und der nachfolgenden Maßnahmen zu ihrer Eindämmung im Frühjahr 2020 ist rasch die Frage nach Auswirkungen der Beschränkung sozialer Kontakte auf die psychische Gesundheit der Bevölkerung aufgekommen. Einsamkeit beschreibt eine wahrgenommene Qualität der eigenen Kontakte und Beziehungen zu anderen Menschen. Zahlreiche Studien haben einen Zusammenhang von Einsamkeit mit somatischen und psychischen Erkrankungen aufgezeigt.

**Ziel:**

Auswertung der Häufigkeit von Einsamkeit und ihrer Beziehung zu Angst- und Depressionssymptomen in der ersten Welle der Pandemie im Mai 2020.

**Methoden:**

Zwischen 2014 und 2019 hat die NAKO-Gesundheitsstudie 205.000 Personen im Alter zwischen 20 und 69 Jahren in 18 Studienzentren in Deutschland rekrutiert und untersucht. Die nachfolgende Zweituntersuchung musste aufgrund der Pandemie im Frühjahr 2020 unterbrochen werden. In dieser Zeit wurde ein COVID-19-bezogener Fragebogen entwickelt und an alle Teilnehmenden verschickt. Ausgewertet wurden die 113.928 Fragebögen, die innerhalb der ersten 30 Tage zurückgeschickt wurden. Einsamkeit wurde mit der 3‑Item UCLA Loneliness Scale, Angst und Depression mit den PHQ-9- und GAD-7-Skalen des Patient Health Questionnaire erhoben.

**Ergebnisse:**

Im Mai 2020 nahmen sich 31,7 % der NAKO-Teilnehmenden als einsam wahr. Frauen und junge Menschen waren häufiger als Männer und ältere Personen betroffen. Mit steigender Wahrnehmung von Einsamkeit nahm der Schweregrad von Depressions- und Angstsymptomen stetig zu. Einsame Personen während der Pandemie hatten bereits zur NAKO-Basisuntersuchung mehr depressive und Angstsymptome angegeben als NAKO-Teilnehmende, die sich in der Pandemie nicht einsam fühlten.

**Schlussfolgerung:**

In der NAKO-Gesundheitsstudie zeigte sich während der ersten Phase der Pandemie eine Zunahme von Einsamkeit und ihr deutlicher Zusammenhang mit schlechterer, psychischer Gesundheit.

## Abstract (en)

**Background:**

Early during the pandemic and the following protective countermeasures, an interest in the consequences of the enacted restriction of social contacts for the mental health of the population arose. Loneliness describes the perceived quality of one’s own contacts and relationships with other individuals. Several prior studies reported associations of loneliness with different somatic and psychiatric disorders.

**Aim:**

To analyse the frequency of loneliness and its association with depression and anxiety symptoms in the first wave of the pandemic in Germany in May 2020.

**Methods:**

The German National Cohort (NAKO) had recruited and examined 205,000 individuals aged 20 to 69 years in 18 study centres across Germany between 2014 and 2019. The follow-up examination was temporarily stopped due to the pandemic between March and July 2020. In this period a COVID-related questionnaire was developed and sent to all participants. We analysed the first 113,928 questionnaires that were sent back within four weeks in May 2020. Loneliness was assessed with the three-item UCLA Loneliness Scale and anxiety and depression symptoms were collected using the PHQ‑9 and GAD‑7 scales from the Patient Health Questionnaire.

**Results:**

Among the NAKO participants, 31.7% reported to be lonely in May 2020. Women and young adults reported more loneliness than men and older adults. With increasing scores of loneliness, the severity of depression and anxiety symptoms also steadily increased. Individuals who were lonely during the pandemic had already reported higher PHQ‑9 and GAD‑7 scores during the baseline examination on average 2.5 years earlier, compared to those who did not feel lonely.

**Conclusions:**

Among participants of the German National Cohort, we observed an increase in loneliness during the first wave of the SARS-CoV‑2 pandemic in spring 2020 and a strong relationship of increasing loneliness with decreasing mental health.

## Einleitung

Im Frühjahr 2020, während der ersten Welle der SARS-CoV-2-Pandemie, wurde von der Bundes- und den Landesregierungen eine Reihe einschneidender Maßnahmen zur Reduktion der rasch ansteigenden Infektionszahlen mit dem Virus SARS-CoV‑2 beschlossen. Dazu zählten nichtpharmakologische Maßnahmen der Hygiene, wie Händewaschen und -desinfektion, die Einhaltung körperlicher Distanz zu anderen Menschen sowie die Schließung von Örtlichkeiten, an denen ein erhöhtes Infektionsrisiko durch die Anwesenheit vieler Menschen mit langen Kontaktzeiten bestand. In der Konsequenz führte dies zur Schließung weiter Bereiche im öffentlichen Leben, den Betrieben, der Bildung und der Kultur, mit dem Ziel soziale Kontakte zu reduzieren [1]. Die Maßnahmen führten zwangsläufig zu mehr sozialer Isolation jedes und jeder Einzelnen (Individualebene).

In der wissenschaftlichen Literatur beschreibt der Begriff „soziale Isolation“ die eher quantitativ erhebbare, geringe Ausprägung eines sozialen Netzes mit einem Mangel an Kontakten [2, 3]. Davon abgegrenzt wird der Begriff „Einsamkeit“ als eine subjektiv wahrgenommene Qualität von Kontakten, die die Diskrepanz zwischen der gewünschten und der tatsächlichen Beziehungsqualität zu anderen Menschen widerspiegelt [3]. In der Literatur finden sich zahlreiche Studien, vor allem für Menschen in mittlerem und hohem Erwachsenenalter, die einen Zusammenhang zwischen Einsamkeit und körperlichen und psychischen Erkrankungen sowie der Mortalität aufzeigen [4–8].

Schon vor der Pandemie ist für die westliche Welt ein ausgeprägtes subjektives Erleben von Einsamkeit beschrieben worden. Deshalb wurde erwartet, dass die Pandemie und ihre weitreichenden, erlassenen Gegenmaßnahmen das Erleben von Einsamkeit verstärken und in diesem Zusammenhang psychische Probleme vermehrt auftreten könnten [9]. Zahlreiche Untersuchungen haben gezeigt, dass während der Pandemie vor allem bei jüngeren Erwachsenen die psychischen Belastungen zugenommen haben [10] und während der ersten Phase von Gegenmaßnahmen hoch blieben [11]. Internationale Studien haben gezeigt, dass Risikofaktoren für das Erleben von Einsamkeit während der Pandemie weibliches Geschlecht und jüngeres Lebensalter sind, aber auch bereits vor der Pandemie bestehende psychische Probleme sowie ein wenig ausgeprägtes soziales Netz und ein geringerer sozioökonomischer Status [12–14]. In einer britischen Studie mit mehreren Messzeitpunkten zeigte sich, dass Menschen mit bereits vor der Pandemie bestehendem deutlichen Einsamkeitserleben dieses während der Einschränkungen noch weitaus gravierender empfanden als vorher. Im Gegensatz dazu erlebten Menschen, die zuvor ein eher geringes Einsamkeitserleben hatten, die Einsamkeit während der Einschränkungen sogar weniger stark als vor den Gegenmaßnahmen [15].

Studien mit Online- oder Befragungspanelteilnehmenden zum Ausmaß von Einsamkeit während der ersten Phase der Pandemie und ihrer Gegenmaßnahmen zeigten anfangs einen geringen Anstieg von Einsamkeit, im weiteren Verlauf jedoch einen leichten Rückgang [16]. Ein Zusammenhang von Einsamkeit und psychischem Belastungserleben ist, ebenfalls auf der Basis von Convenience Samples, berichtet worden [17]. Eine weitere Untersuchung befasste sich mit den Zusammenhängen von Einsamkeit sowie depressiver Symptomatik und Angstsymptomen. Hierbei zeigte sich in einer multivariaten Analyse keine Assoziation von Einsamkeit mit Angst und Depression, jedoch ein Zusammenhang mit einer früheren oder gegenwärtigen psychiatrischen bzw. psychotherapeutischen Behandlung [18].

Ziel der vorliegenden Arbeit ist eine Analyse der Häufigkeit und der alters- und geschlechtsspezifischen Unterschiede im Einsamkeitserleben in einer großen deutschen bevölkerungsbasierten Studie während der ersten Welle der SARS-CoV-2-Pandemie und der angeordneten Gegenmaßnahmen im Frühjahr 2020. Zudem soll die Beziehung zwischen Einsamkeit, Angst und depressiven Symptomen untersucht werden.

## Methoden

Die NAKO-Gesundheitsstudie (kurz: NAKO; [19]) ist eine der sogenannten Megakohorten [20] und hat nach mehrjähriger Planung zwischen 2014 und 2019 mehr als 205.000 Menschen in 18 Studienzentren in 16 deutschen Regionen eingeschlossen. Personen zwischen 20 und 69 Jahren wurden zufällig in den jeweiligen Einwohnermeldeämtern gezogen und zur Studienteilnahme eingeladen [21]. Das Untersuchungsprogramm wurde in 2 unterschiedlich langen Versionen durchgeführt. Etwa 80 % der Teilnehmenden absolvierten eine Standard- (Level 1, L1) und etwa 20 % eine Langversion (Level 2, L2), die im Mittel 4 h bzw. 6 h dauerten. Eine ausführliche Beschreibung der Basisuntersuchung der NAKO findet sich in [22].

Teil der umfangreichen Basisuntersuchungen für alle Teilnehmenden, d. h. sowohl im L1- als auch im L2-Untersuchungsprogramm, war die Erhebung emotionaler Funktionen und psychischer Gesundheit anhand verschiedener Skalen. Der Schweregrad von depressiven und Angstsymptomen wurde mit den Selbstbeantwortungsskalen PHQ‑9 (Patient Health Questionnaire) bzw. GAD‑7 (General Anxiety Disorder Scale) über einen Touchscreenmonitor erfasst. Beide Instrumente sind als Teil des Patient Health Questionnaire [23] validiert und werden vielfach in Bevölkerungs- und Patientenstudien eingesetzt.

Direkt nach Abschluss der Basisuntersuchung begann im Jahr 2019 in allen Studienzentren die Zweituntersuchung der Teilnehmenden, im Mittel etwa 5 Jahre nach der initialen Einladung. Mitte März 2020 musste die Zweituntersuchung aufgrund der Pandemie unterbrochen und die Studienzentren temporär bis Anfang Juli 2020 geschlossen werden. In dieser Situation wurde innerhalb der NAKO kurzfristig die Entscheidung getroffen, allen Teilnehmenden einen COVID-19-bezogenen Fragebogen zu senden mit der Bitte, verschiedene Fragen zu einer möglichen SARS-CoV-2-Infektion sowie zum Umgang mit den zugehörigen Gegenmaßnahmen und zur psychischen Gesundheit während der Pandemie zu beantworten. Der NAKO-COVID-Fragebogen wurde innerhalb von einem Monat entwickelt und ab dem 30.04.2020 an alle NAKO-Teilnehmenden der Basisuntersuchung verschickt. Teilnehmende, von denen eine E‑Mail-Adresse bekannt war, erhielten eine E‑Mail mit einem Link, der das Ausfüllen des Fragebogens online erlaubte. Teilnehmende ohne bekannte E‑Mail-Adresse erhielten den Fragebogen postalisch. Innerhalb von 30 Tagen nach Versendung der ersten Fragebögen, das heißt bis zum Stichtag 29.05.2020, wurden 113.928 NAKO-COVID-Fragebögen beantwortet zurückgesandt. Die Daten aus diesen Fragebögen bilden die Basis der nachfolgenden Auswertung. Die Teilnahmebereitschaft innerhalb dieser ersten 30 Tage variierte zwischen 34 % in Studienzentren im Nordosten und 67 % in denen im Südwesten. Für diese Teilnehmenden wurden die entsprechenden Daten aus der Basisuntersuchung mit denen des NAKO-COVID-Fragebogens zusammengeführt.

Der Fragebogen beinhaltete unter anderem erneut den PHQ‑9 zur Erhebung depressiver Symptome und des Schweregrads depressiver Symptomatik sowie den GAD‑7 zur Erfassung von Angstsymptomen. Zusätzlich wurden 3 Fragen zur subjektiven Wahrnehmung von Einsamkeit aus der 3‑Item-Kurzversion der UCLA Loneliness Scale [2] und eine Frage zur Angst vor einer Coronavirusinfektion aufgenommen. Der übrige Teil des Fragebogens bestand aus Fragen zum Teststatus für SARS-CoV‑2, zu eventuellen COVID-Symptomen, zum Kontaktverhalten während der Pandemie und zu eventuellen Veränderungen in der sozialen Situation und in gesundheitsrelevanten Verhaltensweisen.

Der PHQ‑9 [24] besteht aus 9 Fragen, die sich auf die letzten 2 Wochen beziehen und deren Antwortmuster einen Summenscore von 0 bis 27 Punkten ergeben. Ein Cut-off-Wert von ≥10 Punkten weist auf das Vorliegen einer moderaten bis schweren depressiven Symptomatik hin. Der GAD‑7 [25] enthält 7 Fragen und bezieht sich in der NAKO auf den Zeitraum der letzten 4 Wochen (Score 0–21). Auch hier gilt ein Cut-off-Wert von ≥10 Punkten (moderate bis schwere Angstsymptome) als klinisch relevant. Die Summenscores beider Skalen wurden gemäß dem PHQ-Manual für die Teilnehmenden berechnet, die für beide Instrumente jeweils vollständige Antworten angegeben hatten.

Die Frage zur Angst vor einer Coronavirusinfektion hatte 3 Antwortoptionen (Ja, Nein, Ich weiß nicht) und wurde ergänzt durch gleichlautende Fragen zu Krebs, Herzinfarkt und Naturkatastrophen [26].

Die 3 Fragen zur Einsamkeit aus der Kurzversion der UCLA Loneliness Scale haben eine Likert-skalierte Antwortskala mit 3 Antwortmöglichkeiten (oft = 3, manchmal = 2, selten oder nie = 1). Aus diesen Antworten wurde ein Summenscore von 3 bis 9 gebildet [2, 3]. Bei der Auswertung gibt es 2 Kategorien: keine bis geringe Einsamkeit 3–5 Punkte, moderate bis ausgeprägte Einsamkeit 6–9 Punkte [4]. Eine zusätzliche Frage erhob, ob Einsamkeit im Vergleich zur Zeit vor der Coronapandemie stärker oder weniger stark ausgeprägt bzw. ob die Frage unzutreffend war, da beides für die befragte Person nicht zutraf.

### Statistische Methoden

In der deskriptiven Statistik wurden prozentuale Häufigkeiten, Mittelwerte und Mediane bestimmt. Auf formale Testungen auf Unterschiede in Häufigkeiten oder Mittelwerten wurde verzichtet, da aufgrund der großen Teilnehmendenzahl selbst kleinste, nicht relevante Unterschiede statistische Signifikanz erreichen. Korrelationen zwischen dem Einsamkeitsscore einerseits und dem PHQ-9- bzw. dem GAD-7-Summenscore andererseits wurden mit dem Pearson-Korrelationskoeffizienten bestimmt. Ein multivariables lineares Regressionsmodell wurde eingesetzt, um den Einfluss von soziodemografischen Faktoren und Symptomen von Depressionen und Angst auf Einsamkeit zu analysieren. Dabei bildete der Einsamkeitssummenscore die abhängige Variable und die PHQ-9- und GAD-7-Summenscores sowie Alter, Geschlecht, Schulbildung (keinen/anderen Abschluss, Haupt‑, Realschulabschluss, Fach‑/Abitur) und Studienzentrum die unabhängigen Variablen.

## Ergebnisse

Zwischen dem 30.04. und dem 29.05.2020 wurden 113.928 COVID-Fragebögen von NAKO-Teilnehmenden zurückgesandt. Das Alter der Teilnehmenden entsprach dem in der Basisuntersuchung (im Mittel 50 Jahre; Tab. 1). Es nahmen etwas mehr Frauen als Männer teil (52 % versus 49 %; [10]). Im Mittel erfolgte die Beantwortung des COVID-Fragebogens etwa 2,5 Jahre nach der NAKO-Basisuntersuchung, wobei die Follow-up-Zeit zwischen etwas unter 1 und etwas über 5 Jahren variierte. Der Anteil junger Erwachsener (20–29 Jahre) war mit knapp 10 % deutlich niedriger als derjenige älterer, über 60 Jahre (26,7 %). Etwa 3 Viertel der Antwortenden lebten mit einem/r (Ehe‑)Partner/-in und fast 60 % hatten eine höhere Schulbildung. Summenscores für depressive und Angstsymptome waren auch in der Basisuntersuchung erhoben worden. Sie hatten sich erwartungsgemäß zwischen den Geschlechtern unterschieden. Frauen wiesen im Mittel etwa um einen Punkt höhere Scores für beide Skalen auf als Männer (PHQ-9: 4,2 vs. 3,3 und GAD-7: 3,5 vs. 2,6; Tab. 1).

**Table 1:** 

*Anzahl Teilnehmende 30 Tage (N)*	113.928
*Frauen (%)*	51,80
*Alter (M Jahre)*	49,95
*Alter 20–29 Jahre (%)*	9,14
*Alter 60+ Jahre (%)*	26,69
*Mittlere Follow-up-Zeit (x̅ Jahre)*	2,66
*Schulabschluss (%)*	
Hauptschule	11,26
Mittlere Reife	28,71
(Fach‑)Abitur	58,57
Keinen/anderen Abschluss	1,40
*Mit Partner/in lebend (%)*	76,77
*Depressions- und Angstscores der Basisuntersuchung (2014–2019)*	
Frauen:	
PHQ-9-Score^b^ (M [SD])	4,15 (3,65)
GAD‑7^c^ (M [SD])	3,45 (3,24)
Männer:	
PHQ-9-Score^b^ (M [SD])	3,25 (3,32)
GAD‑7^b^ (M [SD])	2,59 (2,82)

^a^NAKO-Teilnehmer/innen, die den COVID-Fragebogen im Mai 2020, d. h. innerhalb der ersten maximal 30 Tage nach Versendung, ausfüllten. Die aufgeführten Charakteristika sind die vom Zeitpunkt der Basisuntersuchung

^b^Depressionsfragen des Patient Health Questionnaire, minimal 0, maximal 27 Punkte

^c^Generalized Anxiety Disorder Modul, minimal 0, maximal 21 Punkte

In Tab. 2 sind die Ergebnisse zur Einsamkeit und die Angst- und Depressionsscores zu Beginn der Pandemie zusammengefasst. Bei den einzelnen Fragen zur Einsamkeit stand das Fehlen der Gesellschaft anderer im Vordergrund: Fast 80 % der Teilnehmenden gaben im Mai 2020 ein Gefühl „des Fehlens der Gesellschaft anderer“ an. Etwa 35 % fühlten sich „sozial isoliert“ und etwa 30 % gaben ein Gefühl, „außen vor zu sein“, an. Insgesamt waren 31,7 % der NAKO-Teilnehmenden einsam, d. h., sie erreichten einen Wert von 6 Punkten oder mehr auf der UCLA-Einsamkeitsskala. Alle Teilfragen zur Einsamkeit wurden von Frauen häufiger als von Männern bejaht. Insgesamt war der Anteil Einsamer bei Frauen mit 37,4 % deutlich höher als bei Männern mit 25,5 %. Auch berichteten 56,1 % der Frauen und 43,9 % der Männer eine Zunahme der Einsamkeit während der Pandemie.

**Table 2:** 

	Gesamt	Frauen	Männer
*Einsamkeit*			
Gefühl des Fehlens der Gesellschaft anderer^a^ (%)	79,88	83,48	76,00
Gefühl, außen vor zu sein^a^ (%)	29,89	35,09	24,30
Gefühl, sozial isoliert zu sein^a^ (%)	34,83	40,41	28,83
Summenscore „Einsamkeit“^b^ (x̅ [SD])	4,93 (1,61)	5,17 (1,67)	4,68 (1,49)
Anteil „Einsamer“^c^ (%)	31,68	37,44	25,50
Subjektive Zunahme der Einsamkeit während der Pandemie (%)	47,04	56,13	43,87
*Angst vor Lebensereignissen*			
Vor Naturkatastrophe (z. B. Überschwemmung Blitzeinschlag, Sturm) (%)	11,88	15,77	7,71
Vor Krebserkrankung (%)	53,09	58,90	46,86
Vor Herzinfarkt (%)	36,57	36,90	36,21
Vor Coronavirusinfektion (%)	34,51	38,56	30,16
*Depressions- und Angstscores COVID-19-Fragebogen*			
Depression: PHQ-9-Score (M [SD])	4,06 (3,93)	4,59 (4,07)	3,50 (3,69)
Angst: GAD‑7 (M [SD])	3,37 (3,44)	3,91 (3,62)	2,78 (3,12)

^a^Anteil der Antworten „oft“ plus „manchmal“ auf einer 3‑Punkt-Likert-Skala (oft, manchmal, selten oder nie)

^b^Summenscore der Antworten der 3‑Item-Version der UCLA Loneliness Scale (Minimum 3, Maximum 9, Antwortcodierung: „selten oder nie“ = 1, „manchmal“ = 2, „oft“ = 3)

^c^Anteil derjenigen mit moderater bis ausgeprägter Einsamkeit (6–9 Punkte im Einsamkeitsscore)

Gut ein Drittel aller Antwortenden gab Angst vor einer Coronavirusinfektion an (34,5 %). Diese Angst war in der Häufigkeit etwa vergleichbar mit der Angst vor einem Herzinfarkt (36,6 %), niedriger als die Angst vor einer Krebserkrankung (53,1 %), aber deutlich höher als Angst vor einer Naturkatastrophe (11,9 %). Bei allen Ängsten bestand ein Geschlechterunterschied, bei dem Frauen jeweils höhere Angstwerte aufwiesen.

Der PHQ-9-Summenscore für Depressionen lag im Mittel bei beiden Geschlechtern etwa 0,3 Punkte höher als zum jeweiligen Zeitpunkt der Basisuntersuchung. Hingegen nahm der Mittelwert für den GAD-7-Score für Ängste mit 0,45 Punkten bei Frauen stärker zu als bei Männern (0,2 Punkte).

**Figure 1:**
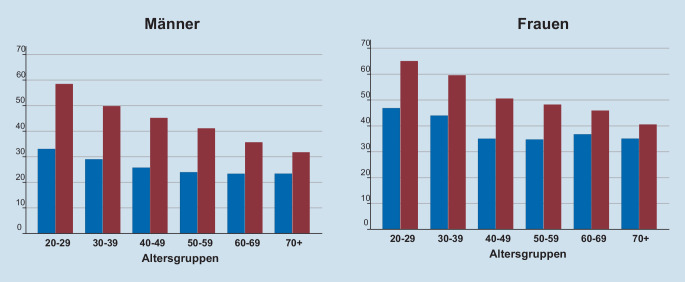

In Abb. 1 ist der Anteil derjenigen, die als „einsam“ entsprechend der UCLA-Einsamkeitsskala kategorisiert werden (blau), nach Alter und Geschlecht dargestellt. In allen Altersgruppen ist der Prozentsatz Einsamer bei Frauen höher. Er sinkt in beiden Geschlechtern mit steigendem Alter. Ein ähnliches Muster findet sich für den Anteil derjenigen, die sich vermehrt einsam während im Vergleich zu vor der Pandemie gefühlt haben (Abb. 1). Auch hier findet sich ein ähnlicher Alterstrend und Frauen sind deutlich stärker betroffen als Männer.

**Figure 2:**
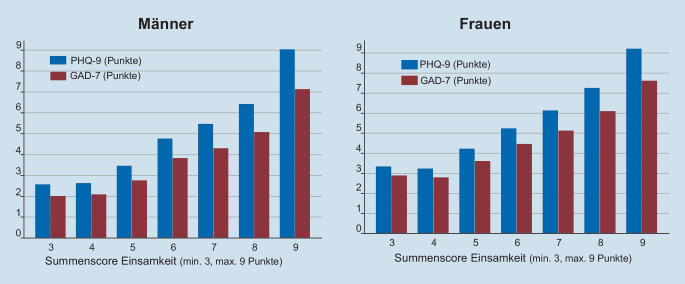

Abb. 2 zeigt den Zusammenhang zwischen depressiven und Angstsymptomen mit dem Einsamkeitsscore. Es findet sich ein deutlicher Zusammenhang mit stetig steigenden, mittleren Depressions- und Angstscores mit jedem Punkt Zunahme im Einsamkeitsscore. Dieser Anstieg beginnt bereits bei 4 bzw. 5 Punkten des Einsamkeitsscores, einem Bereich, der als „nicht einsam“ definiert ist. Die Korrelationen (Pearson-Korrelationskoeffizient) zwischen Einsamkeitsscore und PHQ-9- bzw. GAD-7-Scores betragen 0,38 bzw. 0,36.

Tab. 3 fasst die Koeffizienten aus dem multivariablen linearen Regressionsmodell mit dem Einsamkeitsscore als abhängiger Variable zusammen. PHQ-9-Summenscore, GAD-7-Summenscore und weibliches Geschlecht haben eine positive Assoziation mit dem Einsamkeitsscore, höhere Schulbildung und bestehende Partnerschaft hingegen eine inverse Beziehung. Angst vor einer Coronavirusinfektion ist ebenfalls positiv mit Einsamkeit assoziiert. Der Effekt ist jedoch kleiner als der der erstgenannten 3 Faktoren. Die Assoziation mit dem Studienzentrum war minimal.

**Table 3:** 

Einflussfaktor	Betakoeffizient^a^	95 % Konfidenzintervall	
Alter, pro Jahr	0,0002	−0,0006	0,0009
Weibliches Geschlecht	0,28	0,26	0,31
Schulbildung^b^	−0,04	−0,05	−0,03
In Partnerschaft lebend^c^	−0,13	−0,15	−0,11
PHQ-9-Summenscore, Punkte	0,106	0,10	0,11
GAD-7-Summenscore, Punkte	0,065	0,06	0,07
Angst vor Coronainfektion^c^	0,10	0,08	0,12
Studienzentrum^d^	0,004	0,002	0,006

^a^Unstandardisierte Betakoeffizienten aus einer multivariablen linearen Regression mit dem Einsamkeitsscore als abhängiger und den aufgeführten Einflussfaktoren als gemeinsame, unabhängige Variablen

^b^Schulabschlüsse aufsteigend (keiner/anderen Abschluss, Hauptschulabschluss, Realschulabschluss, Fach‑/Abitur)

^c^Ja vs. Nein

^d^Die Studienzentren wurden aufsteigend nach Nummer sortiert

**Figure 3:**
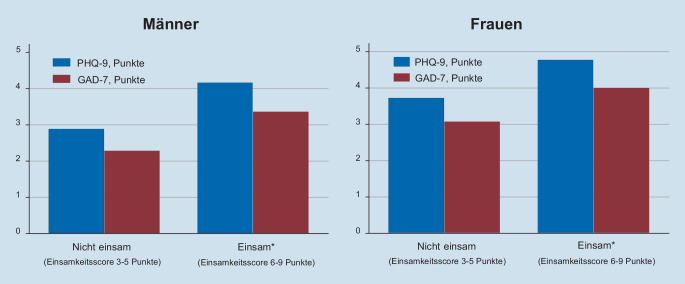

Abb. 3 schließlich zeigt, dass diejenigen, die während der ersten Phase der Pandemie im Frühjahr 2020 „einsam“ waren, bereits zur NAKO-Basisuntersuchung, im Mittel 2,5 Jahre vorher, höhere Mittelwerte bei depressiven und Angstsymptomen aufwiesen. Der Unterschied im PHQ-9-Score bei Männern, zwischen einsamen und nicht einsamen, betrug etwa 1,3 Punkte, der für den GAD-7-Score 1,1. Sehr ähnliche Anstiege bei höheren Ausgangsscores (Tab. 1) fanden sich bei Frauen.

## Diskussion

In der großen, bevölkerungsbasierten NAKO-Gesundheitsstudie waren während der ersten Welle der SARS-CoV-2-Pandemie im Mai 2020 knapp ein Drittel der Studienteilnehmenden einsam. Die Hälfte gab eine Zunahme von Einsamkeit im Vergleich zu Zeiten vor der Pandemie an. Einsamkeit war deutlich mit Depressivität und Angst assoziiert. Frauen waren zu einem höheren Anteil einsam als Männer. Bei Personen mit einer höheren Schulbildung und Teilnehmenden, die in einer Partnerschaft lebten, war der Anteil niedriger als bei jenen mit niedriger Bildung bzw. bei Alleinlebenden. Ein Vergleich mit den Scores der Basisuntersuchung, die im Mittel etwa 2,5 Jahre zuvor stattgefunden hat, ergab, dass diejenigen, die sich im Mai 2020 während der ersten Welle der Pandemie einsam fühlten, bereits zuvor deutlich höhere Depressions- und Angstsymptome aufwiesen als diejenigen, die im Frühjahr 2020 nicht einsam waren.

Unsere Befunde stehen in Einklang mit Studien, die vor der Pandemie durchgeführt wurden und einen Zusammenhang zwischen Einsamkeit und negativer psychischer Gesundheit zeigten [5, 27]. Erste Ergebnisse aus Studien während der COVID-19-Pandemie weisen ebenfalls in diese Richtung. Eine Längsschnittanalyse des Sozioökonomischen Panels (SOEP-CoV) hat einen deutlichen Anstieg von Einsamkeitswerten und depressiver Symptomatik von Messzeitpunkten vor der Pandemie bis zum Frühjahr 2020 gezeigt [28]. Das Ergebnis, dass Merkmale wie höhere Bildung und Partnerschaft einen protektiven Effekt bezüglich Einsamkeit hatten, bietet einen Hinweis darauf, dass Menschen mit besseren wirtschaftlichen bzw. psychosozialen Ressourcen auch besser durch die globale Krise zu kommen scheinen als diejenigen, die sowohl ökonomisch als auch sozial eher benachteiligt sind.

Als große, bevölkerungsbasierte Studie in 16 deutschen Regionen hat die NAKO-Gesundheitsstudie eine Reihe von Vorteilen. Die Teilnehmenden wurden auf Basis von alters- und geschlechtsstratifizierten Zufallsstichproben in den jeweiligen städtischen bzw. regionalen Einwohnermeldeämtern ausgewählt und eingeladen. Der relativ zeitnah zum Inkrafttreten der erlassenen Gegenmaßnahmen verschickte Fragebogen hatte eine hohe Response von knapp 60 % innerhalb der ersten 30 Tage und 82 % insgesamt. Die gleichen Skalen zur Erhebung von Depressions- und Angstsymptomen kamen zur Erstuntersuchung und im COVID-Fragebogen zum Einsatz. Einschränkend muss festgehalten werden, dass diese Auswertung zunächst auf den ersten ca. 114.000 Teilnehmenden beruht, die innerhalb des Mai 2020 antworteten. Bis zum Ende der COVID-Erhebung haben ca. 157.000 Teilnehmer den Fragebogen beantwortet. Die Daten von allen werden allerdings erst im Laufe des Jahres 2021 zur Verfügung stehen. Die Fragen der UCLA-Einsamkeitsskala wurden nur im COVID-Fragebogen gestellt und nicht in der Basisuntersuchung. Deshalb konnte keine Veränderung im Skalenwert berechnet werden und in diesem querschnittlichen Design ist die Richtung des Zusammenhanges zwischen Einsamkeit, depressiven und Angstsymptomen nicht geklärt. Auch spätere denkbare Veränderungen des Skalenwertes, z. B. ein eventueller Rückgang im Sommer 2020 und erneuter Anstieg zum Jahresende, konnten aufgrund des einmaligen Versendens des Fragebogens nicht analysiert werden. Einsamkeit beschreibt in der wissenschaftlichen Literatur eine wahrgenommene Beziehungsqualität zu anderen Menschen. Die Gegenmaßnahmen zur Pandemie im Frühjahr 2020 zielten auf eine Verringerung der Kontakthäufigkeit zwischen Menschen und beeinflussten damit gezielt eine wichtige Komponente von Beziehungsqualität. Das subjektive Empfinden von Einsamkeit ist allerdings unabhängig von den Ursachen einer reduzierten Beziehungsqualität.

Zusammenfassend muss von einer Zunahme von Einsamkeit während der ersten Phase der Pandemie und ihrer Gegenmaßnahmen im Frühjahr 2020 ausgegangen werden. Die vielfach beschriebene Verstärkung der sozialen Ungleichheit durch die Pandemie wirkt sich, wenn die Trends sich bestätigen, auch auf die psychische Gesundheit aus. Das Ergebnis, dass NAKO-Teilnehmende, die sich einsam fühlen, bereits lange vor der Pandemie höhere Scores bei Depression und Angst aufwiesen, zeigt, dass auch Menschen mit geringen bis moderaten depressiven Symptomen rascher subjektiv einsam werden und verweist – bei aller psychometrischen Unterschiedlichkeit – auf das Wechselspiel von Einsamkeit und Depressivität: „Loneliness reflects how you feel about your relationships. Depression reflects how you feel, period“ [29].
